# Prevalence of chronic pelvic pain and associated factors among indigenous women of reproductive age in Ecuador

**DOI:** 10.1186/s12905-024-03189-7

**Published:** 2024-07-04

**Authors:** José Antonio Vargas-Costales, Carmen Yolanda de Las Mercedes Villa Rosero, Suleimy Cristina Mazin, Francisco José Candido-dos-Reis, Antonio Alberto Nogueira, Julio Cesar Rosa-e-Silva, Omero Benedicto Poli-Neto

**Affiliations:** 1 Department of Pharmacology, School of Medicine, Faculty of Medical Sciences, Central University of Ecuador, Quito, Ecuador; 2 Department of Anesthesiology, School of Medicine, Faculty of Medical Sciences, Universidad Central del Ecuador, Quito, Ecuador; 3 Department of Obstetrics and Gynecology, Laboratory for Translational Data Science, Ribeirão Preto Medical School of the University of São Paulo USP, Bandeirantes Avenue. Monte Alegre. Ribeirão Preto, Ribeirão Preto, SP 3900, 049-900 Brazil; 4 Laboratory for Translational Data Science, CNPq (Conselho Nacional de Desenvolvimento Científico e Tecnológico), Brasília, Brazil

**Keywords:** Dyspareunia, Ecuador, Indigenous, Kichwa, Non-cyclic pelvic pain, Prevalence, Primary dysmenorrhoea, Risk factor

## Abstract

**Background:**

Chronic pelvic pain is a common disease that affects approximately 4% of women of reproductive age in developed countries. This number is estimated to be higher in developing countries, with a significant negative personal and socioeconomic impact on women. The lack of data on this condition in several countries, particularly those in development and in socially and biologically vulnerable populations such as the indigenous, makes it difficult to guide public policies.

**Objectives:**

To evaluate the prevalence of chronic pelvic pain (dysmenorrhea, dyspareunia, non-cyclical pain) and identify which variables are independently associated with the presence of the condition in indigenous women from Otavalo-Ecuador.

**Design:**

A cross-sectional study was carried out including a sample of 2429 women of reproductive age between 14 and 49 years old, obtained from April 2022 to March 2023. A directed questionnaire was used, collected by bilingual interviewers (Kichwa and Spanish) belonging to the community itself; the number of patients was selected by random sampling proportional to the number of women estimated by sample calculation. Data are presented as case prevalence, odds ratio, and 95% confidence interval, with *p* < 0.05.

**Results:**

The prevalence of primary dysmenorrhea, non-cyclic pelvic pain, and dyspareunia was, respectively, 26.6%, 8.9%, and 3.9%.all forms of chronic pain were independently associated with each other. Additionally, dysmenorrhoea was independently associated with hypertension, intestinal symptoms, miscegenation, long cycles, previous pregnancy, use of contraceptives and pear body shape. Pain in other sites, late menarche, exercise, and pear body shape were associated with non-cyclic pelvic pain. And, urinary symptoms, previous pregnancy loss, miscegenation, and pear body shape were associated with dyspareunia.

**Conclusion:**

The prevalence of primary dysmenorrhea and non-cyclical chronic pelvic pain was notably high, in contrast with the frequency of reported dyspareunia. Briefly, our results suggest an association between dysmenorrhoea and conditions related to inflammatory and/or systemic metabolic disorders, including a potential causal relationship with other manifestations of pelvic pain, and between non-cyclical pelvic pain and signs/symptoms suggesting central sensitization. The report of dyspareunia may be influenced by local cultural values and beliefs.

**Supplementary Information:**

The online version contains supplementary material available at 10.1186/s12905-024-03189-7.

## Introduction

Chronic pelvic pain (CPP) is a complex and debilitating medical condition that affects people around the world regardless of gender or age, although it is most frequently reported in people assigned female at birth (women henceforth) and throughout their reproductive years [1]. Most recent estimates suggest a prevalence between 5% and 26% [2]. However, it seems to have significant differences between developing and developed countries [3].

A significant portion of women sufferers has a detrimental impact on their life trajectory, with reduced quality of life, depressive symptoms, anxiety [4], decreased workplace productivity [5], and diminished sexual satisfaction [6]. Additionally, it is associated with a significant economic burden on women’s lives and the community as a whole [7, 8]. While it is possible to alleviate the symptoms of patients to a great extent, and even suppress them, recurrence frequently occurs over time. This is a source of dissatisfaction not only for the patients themselves but also for the healthcare professionals who care for them. Treatment often is not curative, and personalised care within a multidisciplinary perspective is very important [9].

The International Association for the Study of Pain (IASP) defines chronic pelvic pain as “*chronic or persistent pain perceived in structures related to the pelvis of either men or women. It is often associated with negative cognitive, behavioural, sexual, and emotional consequences and with symptoms suggestive of lower urinary tract, sexual, bowel, pelvic floor, or gynaecological dysfunction*”. In this definition, continuous or recurrent pain such as dysmenorrhea and dyspareunia are also included (https://www.iasp-pain.org/publications/free-ebooks/classification-of-chronic-pain-second-edition-revised/, accessed in 10/01/2024). The ReVITALize, an initiative led by the Royal College of Obstetricians and Gynaecologists (RCOG), proposes a very similar definition (https://www.acog.org/practice-management/health-it-and-clinical-informatics/revitalize-gynecology-data-definitions, accessed in 10/01/2024).

Dysmenorrhoea is the most common cause of cyclic CPP. The prevalence varies between 50% and 75%, with approximately 20% of women reporting severe associated pain [10]. It is defined as a painful menstrual cycle with a cramping sensation in the lower abdomen immediately before or during the menstruation period. Usually, it is classified as primary or secondary dysmenorrhoea. Each of them has its own characteristics. Primary dysmenorrhoea typically begins near menarche, as soon as ovulatory cycles are established, and is not associated with any obvious pelvic pathology. Secondary dysmenorrhoea typically starts some time after menarche and is associated with an underlying medical condition, appearing even in a woman’s fourth or fifth decade of life. Primary dysmenorrhoea has become a progressively growing area of interest in recent years [11]. Firstly, because it has a significant impact on the lives of young women [12], being associated with lower quality of life [13], worse psychological well-being [14], poorer sleep quality [15], inattention and problems with hyperactivity and impulsivity [16], and lower frequency and academic performance [17]. Secondly, because the literature has shown evidence that dysmenorrhoea may be a general risk factor for future chronic pain, both pelvic and extra-pelvic [18, 19], as well as other conditions such as ischemic heart disease [20].

Painful sexual intercourse, or dyspareunia, is also a common and overlooked health issue among women. It has been reported by approximately 8–26% of sexually active women [3, 21], of whom one-quarter experienced symptoms very often or always for ≥ 6 months, usually linked to poorer sexual, physical, relational, and mental health [22], including depression, anxiety, low self-esteem, negative body image [23], and symptom hypervigilance [24]. A precise and widely accepted classification of the condition is lacking, which results in a poorly understood pathophysiology. Generally, it can also be divided into superficial and deep dyspareunia depending on the depth within the vagina where it is perceived [25]. With regard to the cause, it may be associated with specific pathologies such as endometriosis [26], but often does not appear to be clearly linked to a disease, but rather associated with conditions such as sexual abuse, fear of physical abuse [27], or even partners’ cognitive responses [28].

Over the past two decades, the number of publications considering the topic has significantly increased. Evidence has suggested that the epidemiology, perception, tolerance and treatment of pain are influenced by multiple biological, psychological, emotional, psychosocial and cognitive factors, as well as those related to racial, cultural and ethnic origin [29, 30]. However, the paucity of pain studies among the indigenous people, particularly of those from South America is yet relevant. Only some studies have reported it, as signs and symptoms of temporomandibular disorders among Tsachila and Quichua patients in Ecuador [31], headache and migraine among the Tzeltal Maya in Mexico, Kamayur´a in Brazil, and Uru-Chipaya in Bolivia [32], and Peru [33], and pain management in Amazon indigenous in Brazil [34]. Actually, there are still gaps in knowledge and understanding on this subject in indigenous population whose number in the world is estimated to be around 370–500 million people. While this number constitutes about 5–6% of the global population, it corresponds to 15% of those living in extreme poverty and has a life expectancy roughly 20 years lower than the non-indigenous population [35]. There is also a higher incidence of diseases such as diabetes, cancer, mental disorders, tuberculosis among indigenous populations [36]. Access to healthcare services has been one of the most significant social determinants in this population. They face various barriers, including living in remote areas, having limited financial resources, and having distinct cultural values [37]. And throughout history, they have suffered deep discrimination based on ethnicity, poverty, and language compared to their non-indigenous counterparts, possibly stemming from a history of colonisation, discrimination, marginalisation, subjugation, and dispossession. This vulnerability became even more evident during the COVID-19 pandemic [38]. A high prevalence of chronic pain has been reported in the indigenous population [39], but there is, in fact, limited evidence of the true impact of this condition on the health of this population [40]. Similarly, pain management has generally been guided by their own cultural values [34], although these findings may be described in more details in the near future [41]. This same aspect of vulnerability has also been observed in Ecuador among the Kichwa population, particularly in young women of reproductive age [42].

The Kichwa ethnic group is situated in both the Sierra and the Amazon regions, spanning across 14 distinct communities. Among these is the Kichwa people of Otavalo, an indigenous group residing in the Otavalo region of Ecuador. Over the centuries, dating back to the pre-Inca era, they have preserved their cultural heritage. This population is renowned for their profound connection with nature, communal solidarity, reverence for elders who hold ancestral wisdom and healthcare knowledge, spiritual beliefs, and diligent work ethic [43]. The experience of pain among the Kichwa is intricate, and their strategies for comprehension and management are sophisticated and different from a typical urban population. Emotions, life events, spiritual beliefs, and other cultural elements significantly influence the perception, diagnosis, coping, and treatment of pain [44]. Given the unique circumstances of indigenous women, the widespread global prevalence of chronic pelvic pain, and its detrimental impact on quality of life, this study aims to ascertain the prevalence of chronic pelvic pain and its subtypes (primary dysmenorrhea, non-cyclic pelvic pain, and persistent dyspareunia) within this population. Additionally, the study seeks to elucidate the associated factors in this population.

## Materials and methods

### Study design

A cross-sectional study was carried out that included women of reproductive age between 14 and 49 years of age, between April 2022 and March 2023 in Otavalo, Ecuador, which has a population largely made up of the indigenous group. The study was approved by the Ethics Committee for Research in Human Beings of the Central University of Ecuador. The human research parameters of the Declaration of Helsinki were met, including the signing of the respective informed consent or assent, which were applied in Kichwa language, respecting the culture of the community and allowing a better understanding of the text. Women of reproductive age between 14 and 49 years old at the date of the interview, residing in the Otavalo canton, were included. Pregnant and lactating women were not eligible to participate.

### Interviewer training and data management

Four interviewers who were not linked to the municipality’s health care programs were trained. The interviewers were trained and supervised in-person and coordinated by the researcher responsible for the study. Doubts were resolved through virtual meetings through the web or in-person when necessary.

The collection of information was carried out with the assistance of trained bilingual interviewers (Kichwa and Spanish), belonging to the community, knowledgeable about its habits and values. Additionally, the interviewers were trained to be able to resolve any doubts that the women interviewed may have. Before the start of the field work, a pre-test of the questionnaire was carried out through an interview with 50 women living in the area on questions selected by random draw.

All forms were transferred to a Research Electronic Data Capture (REDCap) electronic database [45] and confirmed in a second analysis by a second researcher independent of the interviewer.

### Research location

The study was carried out in the Otavalo canton, belonging to the Imbabura province, located in the northern inter-Andean region of Ecuador. According to the 2022 census, it had a population of 114,303 inhabitants, of which 51.9% (59,288) were women, with an estimated percentage of women of reproductive age (between 14 and 49 years) of 48.4% of the total of women, the majority of whom (97.5%) are indigenous and mestizos (https://citypopulation.de/en/ecuador/admin/imbabura/1004__otavalo/, accessed in 10/01/2024).

### Data source and measurement methods

The information was obtained through an interview conducted in a confidential and private environment, during preventive medicine appointments. The choice of the participant was made through a stratified random sampling proportional to the number of residences in the parish and the number of women estimated by the size of the sample (Supplementary Table 1).

Data collection was facilitated by and conducted in compliance with the World Endometriosis Research Foundation Endometriosis Phenome and Biobanking Harmonisation Project (WERF EPHect), whose aim is “to enable large-scale, cross-centre, epidemiologically robust research into the causes of endometriosis, novel diagnostic methods, and better treatments through the development of an international consensus on: standardised detailed clinical and personal phenotyping (phenome) data to be collected from women with endometriosis and controls, to improve patient and disease characterisation; and standard operating procedures (SOPs) for banking of biological samples from women with endometriosis and controls, with respect to collection, transport, processing, and long-term storage” [46–49]. We used the Spanish version of the questionnaire, for which the respective access was obtained (https://ephect.org/tools/patient-questionnaire). Semantic adaptations were validated through a preliminary test.

Study data were collected and managed using REDCap electronic data capture tools hosted at Ribeirão Preto Medical School of the University of São Paulo, Brazil [https://redcap.fmrp.usp.br/]. REDCap (Research Electronic Data Capture) is a secure, web-based software platform designed to support data capture for research studies, providing (1) an intuitive interface for validated data capture; (2) audit trails for tracking data manipulation and export procedures; (3) automated export procedures for seamless data downloads to common statistical packages; and (4) procedures for data integration and interoperability with external sources [50, 51].

### Sample size

The sample size, according to the study design, was calculated considering a prevalence of 10% (refs), with a confidence level of 95% and a relative error of 12% [52]. Thus, it was defined as a sample of at least 2401 women of reproductive age, between 14 and 49 years old, who reside in the Otavalo canton. There was no age stratification.

### Bias and minimization methods

Potential biases were associated with data collection, data transfer to the database, and data analysis. To minimise them, we proceeded as follows: 5% of the women in each parish were re-interviewed so that the accuracy of the information provided could be verified. Any disagreement was confirmed with the participant and the information confirmed in the last interview was considered. All forms were transferred to the REDCap and confirmed in a second analysis by a second researcher independent of the interviewer. Data analysis was performed by one of the researchers without access to the interviews and by a statistician with no prior knowledge of the clinical data or characterization data of the participants.

### Primary outcomes and characterisation features

The primary outcome was chronic pelvic pain. We also conducted a separate analysis for each of the subtypes: primary dysmenorrhea, non-cyclic pelvic pain, and persistent dyspareunia. For evaluating this last, we only considered women who had previously had sexual intercourse.

We consider the following features as independent variables: age, education level (higher: post-secondary education, or certain college or professional schools, university, graduate education; intermediate: secondary/junior education or bachelor’s degree; minimal or none: primary education or none), body mass index (BMI), obesity (BMI > 30), exercise (minimum of 2 h per week), body type (pear, straight, hourglass, apple), pain catastrophizing scale [53], ethnicity (unmixed or mixed), hormone usage, menstrual irregularity on the last 3 months, duration of menstrual flow, volume of bleeding, length of the menstrual cycle, coitarche, previous pregnancy, subfertility, age of the first pregnancy, pregnancy while teenage, number of pregnancies, caesarean section, previous pregnancy loss (spontaneous abortion, stillbirth, induced abortion, ectopic pregnancy), alcoholism according alcohol misuse concept (https://www.nhs.uk/conditions/alcohol-misuse/*)*, previous or current smoking, anxiety, depression, diabetes, hypertension, polycystic ovary syndrome, intestinal symptoms, urinary symptoms, migraine, endometriosis, previous abdominal or pelvic surgery, familial history of pelvic pain, other pains (low back, joint, ovulation, legs, urinating, defecating).

### Statistical methods

All exploratory and statistical analyses were implemented in Python. Statsmodels, SciPy, and Scikit-learn were the packages utilised.

Initially, an exploratory data analysis was carried out, considering the measures of central position (mean and median) and dispersion (standard deviation and range). For the qualitative variables, the absolute and relative frequencies were estimated. The Shapiro–Wilk test was used to verify normality. Student’s t test was used for univariate analyses to compare means between the binary outcomes: dysmenorrhea, non-cyclic pelvic pain, and dyspareunia. Chronic pelvic pain was considered to be present in cases where any of the subtypes were evident. Fisher’s exact test or Chi-square test for qualitative variables was used when appropriate. For analysing dyspareunia, only women previously ever sexually active were included.

Before conducting multivariate analysis, a correlogram was created using bivariate Spearman correlation to assess monotonic relationships (whether linear or not) between each pair of variables. Those with a correlation coefficient greater than 0.60 were either avoided or cautiously considered.

To determine which variables were associated with each of the results, log-binomial regression models were constructed. We initially employed three different strategies for variable selection, in addition to the background acknowledgment: including variables with differences between groups in univariate analysis (*p* < 0.20), forward and backward stepwise selection. The model that best fit our data was the full model followed by augmented backward elimination. Ultimately, all models were adjusted by age, race, and hormone usage. For the choice of the best of them, the following principles were considered: Log-likelihood, Bayesian (BIC), and Akaike information criterion (AIC). We presented the results using prevalence ratio and confidence interval. We assessed the linearity assumption and checked for separation, and influential outliers (no issues were found). We used variance inflation factor (VIF) to diagnose collinearity and after analysing its impact, we progressively eliminated variables with a value greater than 5. To assess the calibration of the models, we utilised the Brier score. We used the area under the receiver operating characteristic (ROC) curve to assess the discrimination of the model.

## Results

The final sample included 2,429 women of childbearing age between 14 and 49 years of age residing in Otavalo. The prevalence of chronic pelvic pain was 33.0% (*n* = 802). Considering the subtypes, the prevalence of primary dysmenorrhea, non-cyclic pelvic pain, and dyspareunia was, respectively, 26.6%, 8.9%, and 3.9%. Table 1 shows the characterization of the interviewed participants and the results of the univariate analysis.

**Table 1:** Characteristics of the interviewed participants according to the primary outcomes

Features		Primary dysmenorrhoea 26.6% (647/2429)			Non-cyclic pelvic pain 8.9% (216/2429)			Dyspareunia 3.9% (80/2061)		
		Yes	No	*p*-value	Yes	No	*p*-value	Yes	No	*p*-value
Age (years), mean ± sd		24.5 ± 8.3	28.6 ± 9.3	< 0.001	30.0 ± 9.4	27.3 ± 9.2	< 0.001	31.5 ± 8.1	28.6 ± 9.1	0.006
Education level, % (n)	minimum	12.8 (83/647)	25.6 (456/1782)	< 0.001	25.9 (56/216)	21.8 (483/2213)	0.112	17.5 (14/80)	24.7 (510/2061)	0.201
	medium	66.5 (430/647)	57.5 (1025/1782)	--	53.2 (115/216)	60.6 (1340/2213)	--	57.5 (46/80)	56.4 (1163/2061)	--
	high	20.7 (134/647)	16.9 (301/1782)	--	20.8 (45/216)	17.6 (390/2213)	--	25.0 (20/80)	18.8 (388/2061)	--
BMI (Kg.m^-2^), mean ± sd		24.7 ± 4.8	25.3 ± 4.7	0.007	26.3 ± 5.4	25.0 ± 4.7	< 0.001	26.7 ± 4.1	25.4 ± 4.9	0.021
Obesity (BMI > 30), % (n)		8.7 (56/647)	13.1 (234/1782)	0.002	18.5 (40/216)	11.3 (250/2213)	0.003	17.5 (14/80)	13.1 (271/2061)	0.243
Exercise, % (n)		70.6 (457/647)	59.9 (1067/1782)	< 0.001	16.2 (129/216)	63.0 (1395/2213)	0.339	72.5 (58/80)	60.9 (1255/2061)	0.046
Body type, % (n)	pear	29.2 (189/647)	53.7 (957/1782)	< 0.001	31.5 (68/216)	48.7 (1078/2213)	< 0.001	28.7 (23/80)	48.3 (995/2061)	< 0.001
	straight	23.8 (154/647)	18.1 (322/1782)	--	26.4 (57/216)	18.9 (419/2213)	--	18.8 (15/80)	19.4 (399/2061)	--
	hourglass	30.3 (196/647)	11.8 (210/1782)	--	23.1 (50/216)	16.1 (356/2213)	--	37.5 (30/80)	14.8 (306/2061)	--
	apple	16.7 (108/647)	16.4 (293/1782)	--	19.0 (41/216)	16.3 (360/2213)	--	15.0 (12/80)	17.5 (361/2061)	--
Catastrophizing, mean ± sd		10.8 ± 11.1	7.3 ± 9.0	< 0.001	11.4 ± 12.1	7.9 ± 9.4	< 0.001	13.8 ± 12.4	8.0 ± 9.6	< 0.001
Unmixed ethnicity % (n)		40.2 (260/647)	49.0 (873/1782)	< 0.001	42.1 (91/216)	47.1 (1042/2213)	0.175	37.5 (30/80)	45.9 (946/2061)	0.169
Menarche (years), mean ± sd		13.3 ± 1.4	13.1 ± 1.5	0.002	12.9 ± 1.4	13.2 ± 1.5	0.023	13.0 ± 1.4	13.1 ± 1.5	0.484
Hormone usage, % (n)		3.4 (22/647)	6.2 (111/1782)	0.006	5.6 (12/216)	5.5 (121/2213)	0.876	3.8 (3/80)	6.3 (130/2061)	0.480
Menstrual irregularity, % (n)		24.1 (156/647)	25.5 (454/1782)	0.525	30.6 (66/216)	24.6 (544/2213)	0.059	33.8 (27/80)	25.3 (521/2061)	0.091
Menstrual flow (days), mean ± sd		4.9 ± 1.9	4.7 ± 1.9	0.006	5.2 ± 2.9	4.7 ± 1.7	< 0.001	4.9 ± 2.1	4.7 ± 1.9	0.360
Prolonged menstrual bleeding, % (n)		1.9 (12/647)	1.7 (30/1782)	0.728	4.2 (9/216)	1.5 (33/2213)	0.010	3.8 (3/80)	1.8 (38/2061)	0.196
Average bleeding, % (n)	spotting	26.6 (172/647)	15.5 (276/1782)	< 0.001	23.6 (51/216)	17.9 (397/2213)	0.015	27.5 (22/80)	16.9 (349/2061)	0.050
	light	49.8 (322/647)	59.0 (1052/1782)	--	46.3 (100/216)	57.6 (1274/2213)	--	43.8 (35/80)	57.4 (1184/2061)	--
	moderate	21.0 (136/647)	23.6 (420/1782)	--	27.3 (59/216)	22.5 (497/2213)	--	26.2 (21/80)	23.4 (482/2061)	--
	heavy	2.6 (17/647)	1.9 (34/1782)	--	2.8 (6/216)	2.0 (45/2213)	--	2.5 (2/80)	2.2 (46/2061)	--
Menstrual cycle length, % (n)	normal	88.4 (572/647)	85.0 (1515/1782)	< 0.001	81.9 (177/216)	86.3 (1910/2213)	0.195	83.8 (67/80)	85.5 (1763/2061)	0.891
	< 24 days	4.9 (32/647)	3.1 (56/1782)	--	5.1 (11/216)	3.5 (77/2213)	--	3.8 (3/80)	3.6 (75/2061)	--
	> 38 days	6.6 (43/647)	11.8 (211/1782)	--	13.0 (28/216)	10.2 (226/2213)	--	12.5 (10/80)	10.8 (223/2061)	--
Ever sexually active, % (n)		85.0 (550/647)	89.3 (1591/1782)	0.006	95.4 (206/216)	87.4 (1935/2213)	< 0.001	100.0 (80/80)	100.0 (2061/2061)	--
Previous pregnancy, % (n)		41.6 (269/647)	67.7 (1206/1782)	< 0.001	72.2 (156/216)	59.6 (1319/2213)	< 0.001	78.8 (63/80)	68.2 (1406/2061)	0.049
Subfertility, % (n)		1.4 (9/647)	0.6 (11/1782)	0.075	2.3 (5/216)	0.7 (15/2213)	0.027	5.0 (4/80)	0.8 (16/2061)	0.006
Age of first pregnancy (years), mean ± sd		20.3 ± 3.8	20.1 ± 4.1	0.459	19.9 ± 4.0	20.2 ± 4.0	0.329	19.8 ± 3.5	20.2 ± 4.1	0.434
Teenage pregnancy, % (n)		19.8 (128/647)	35.4 (631/1782)	< 0.001	38.4 (83/216)	30.5 (676/2213)	0.021	38.8 (31/80)	35.1 (723/2061)	0.551
Number of pregnancies, median (Q1-Q3)		0 (0 to 1)	1 (0 to 2)	< 0.001	2 (0 to 3)	1 (0 to 2)	< 0.001	2 (1 to 3)	1 (0 to 2)	0.025
Cesarean section, % (n)		6.2 (40/647)	7.6 (136/1782)	0.250	12.0 (26/216)	6.8 (150/2213)	0.008	8.8 (7/80)	8.1 (167/2061)	0.834
Cesarean section, median (Q1-Q3)		0 (0 to 0)	0 (0 to 0)	0.372	0 (0 to 0)	0 (0 to 0)	0.002	0 (0 to 0)	0 (0 to 0)	0.955
Previous pregnancy loss, % (n)		3.1 (20/647)	3.6 (65/1782)	0.617	7.4 (16/216)	3.1 (69/2213)	0.003	13.8 (11/80)	3.5 (73/2061)	< 0.001
Pregnancy loss, median (Q1-Q3)		0 (0 to 0)	0 (0 to 0)	0.723	0 (0 to 0)	0 (0 to 0)	0.001	0 (0 to 0)	0 (0 to 0)	< 0.001
Alcoholism, % (n)		0.8 (5/647)	0.7 (12/1782)	0.786	0.5 (1/216)	0.7 (16/2213)	1.000	0.0 (0/80)	0.8 (17/2061)	--
Smoking, % (n)		0.5 (3/647)	0.4 (7/1782)	0.732	0.5 (1/216)	0.4 (9/2213)	0.607	1.2 (1/80)	0.4 (9/2061)	0.317
Anxiety, % (n)		0.6 (4/647)	0.4 (8/1782)	0.532	0.9 (2/216)	0.5 (10/2213)	0.290	0.0 (0/80)	0.5 (11/2061)	--
Depression, % (n)		0.6 (4/647)	1.0 (17/1782)	0.620	0.5 (1/216)	0.9 (20/2213)	1.000	1.2 (1/80)	0.9 (18/2061)	0.516
Diabetes, % (n)		0.6 (4/647)	0.5 (9/1782)	0.755	0.5 (1/216)	0.5 (12/2213)	1.000	1.2 (1/80)	0.6 (12/2061)	0.391
Hypertension, % (n)		1.4 (9/647)	0.7 (12/1782)	0.133	1.4 (3/216)	0.8 (18/2213)	0.426	0.0 (0/80)	1.0 (21/2061)	--
Polycystic ovary syndrome, % (n)		1.9 (12/647)	2.0 (36/1782)	0.87	4.2 (9/216)	1.8 (39/2213)	0.034	2.5 (2/80)	2.1 (44/2061)	0.689
Intestinal symptoms, % (n)		51.5 (333/647)	29.6 (528/1782)	< 0.001	44.0 (95/216)	34.6 (766/2213)	0.007	51.2 (41/80)	34.4 (709/2061)	0.003
	diarrhea	2.6 (17/647)	3.1 (56/1782)	0.592	6.0 (13/216)	2.7 (60/2213)	0.012	2.5 (2/80)	3.2 (65/2061)	1.000
	constipation	4.2 (27/647)	3.9 (69/1782)	0.725	5.1 (11/216)	3.8 (85/2213)	0.359	7.5 (6/80)	3.9 (80/2061)	0.134
Urinary symptoms, % (n)		35.1 (227/647)	27.8 (495/1782)	0.001	44.0 (95/216)	28.3 (627/2213)	< 0.001	66.2 (53/80)	29.3 (604/2061)	< 0.001
Migraine, % (n)		1.1 (7/647)	1.1 (19/1782)	1.000	1.9 (4/216)	1.0 (22/2213)	0.283	1.2 (1/80)	1.1 (23/2061)	0.601
Endometriosis, % (n)		0.3 (2/647)	0.2 (4/1782)	0.66	0.9 (2/216)	0.2 (4/2213)	0.093	0.0 (0/80)	0.2 (5/2061)	--
Previous abdominal/pelvic surgery, % (n)		17.8 (115/647)	23.3 (416/1782)	0.003	31.5 (68/216)	20.9 (463/2213)	0.001	36.2 (29/80)	23.6 (486/2061)	0.016
Primary dysmenorrhoea, % (n)		--	--	--	41.7 (90/216)	25.2 (557/2213)	< 0.001	53.8 (43/80)	24.6 (507/2061)	< 0.001
Non-cyclic pelvic pain, % (n)		13.9 (90/647)	7.1 (126/1782)	< 0.001	--	--	--	30.0 (24/80)	8.8 (182/2061)	< 0.001
Dyspareunia, % (n)		6.6 (43/647)	2.1 (37/1782)	< 0.001	11.1 (24/216)	2.5 (56/2213)	< 0.001	--	--	--
Familial history of pelvic pain, % (n)		6.6 (43/647)	6.8 (121/1782)	1.000	10.6 (23/216)	6.4 (141/2213)	0.022	10.0 (8/80)	6.4 (131/2061)	0.240
Other pains, % (n)		8.0 (52/647)	12.7 (227/1782)	0.001	16.7 (36/216)	11.0 (243/2213)	0.018	5.0 (4/80)	12.8 (264/2061)	0.038

*Notes* Q1 = first quartile (25%), Q3 = third quartile (75%)

Among the women with primary dysmenorrhoea, 13.9% and 6.6% had, respectively, non-cyclic pelvic pain and dyspareunia. Among those with non-cyclic pelvic pain, 41.7% and 11.1% had, respectively, primary dysmenorrhoea and dyspareunia. Finally, among those with dyspareunia, 53.8% and 30.0% had, respectively, primary dysmenorrhoea and non-cyclic pelvic pain.

Non-cyclical pelvic pain, dyspareunia, and intestinal symptoms were positively associated with primary dysmenorrhea. We observed the same direction of the association with hypertension. On the other hand, indigenous people without miscegenation (unmixed), long cycles, previous pregnancy (and if in adolescence), use of contraceptives and pear body shape were negatively associated with this outcome. Table 2 shows these results in detail.

**Table 2:** Features independently associated with primary dysmenorrhoea in the population predominantly indigenous of Otavalo, Ecuador

Features	PR	95% CI	adjusted *p*-value	VIF
Hypertension	1.8	0.99–3.10	0.055	1.0
Dyspareunia	1.8	1.34–2.46	< 0.001	1.1
Non-cyclic pelvic pain	1.4	1.13–1.67	0.001	1.1
Intestinal symptoms	1.3	1.17–1.47	< 0.001	1.5
Catastrophizing	1.0	1.01–1.02	< 0.001	1.7
Teenage pregnancy	0.9	0.76–1.04	0.156	2.1
Unmixed ancestry	0.8	0.68–0.83	< 0.001	1.6
Cycle length > 38 days	0.8	0.66-1.00	0.053	1.1
Previous pregnancy	0.5	0.42–0.54	< 0.001	3.1
Hormone usage	0.8	0.63–1.10	0.190	1.1
Pear body shape	0.5	0.45–0.56	< 0.001	1.6

*Notes* PR = prevalence ratio; CI = confidence interval; VIF: variance inflation factor. Menarche and age were removed from the model due to their respective variance inflation factors (VIF) exceeding 5, specifically 13.3 and 7.0. Brier Score: 0.162. Area under ROC curve: 0.756

Dyspareunia, pain at ovulation, primary dysmenorrhoea, and urinary symptoms were positively associated with non-cyclic pelvic pain. On the other hand, late menarche, exercise and pear body shape were negatively associated with this outcome. Table 3 shows these results in detail.

**Table 3:** Factors independently associated with non-cyclical pelvic pain in the population predominantly indigenous of Otavalo, Ecuador

Features	PR	95% CI	adjusted *p*-value	VIF
Dyspareunia	2.1	1.52–2.88	< 0.001	1.1
Pain at ovulation	1.7	1.13–2.63	0.011	1.1
Muscle/joint pain	1.6	1.02–2.51	0.040	1.1
Primary dysmenorrhoea	1.4	1.19–1.68	< 0.001	1.4
Urinary symptoms	1.3	1.08–1.47	0.004	1.5
Teenage pregnancy	1.2	0.99–1.37	0.062	1.6
Catastrophizing	1.0	1.00-1.02	0.024	1.8
Age	1.0	0.96–0.97	< 0.001	4.5
Hormone usage	0.8	0.55–1.07	0.114	1.1
Unmixed ancestry	0.8	0.66–0.87	< 0.001	1.8
Exercise	0.7	0.57–0.74	< 0.001	2.4
Pear body shape	0.6	0.49–0.66	< 0.001	1.8

*Notes* PR = prevalence ratio; CI = confidence interval; VIF: variance inflation factor. Menarche and previous pregnancy were removed from the model due to their respective variance inflation factors (VIF) exceeding 5, specifically 15.9 and 6.0. Brier Score: 0.083. Area under ROC curve: 0.607

Urinary symptoms, pregnancy loss, primary dysmenorrhoea, and non-cyclic pelvic pain were positively associated with dyspareunia. On the other hand, late menarche, hormone usage and pear body shape were negatively associated with this outcome. Table 4 shows these results in detail.

**Table 4:** Factors independently associated with dyspareunia in the population predominantly indigenous of Otavalo, Ecuador

Features	PR	95% CI	adjusted *p*-value	VIF
Pregnancy loss	2.2	1.46–3.17	< 0.001	1.1
Urinary symptoms	1.7	1.38–2.08	< 0.001	1.5
Primary dysmenorrhoea	1.6	1.29–2.09	< 0.001	1.3
Non-cyclic pelvic pain	1.6	1.24–2.13	< 0.001	1.2
Catastrophizing	1.0	1.00-1.01	0.257	1.8
Age	1.0	0.94–0.95	< 0.001	3.5
Race	0.7	0.56–0.83	< 0.001	1.7
Hormone usage	0.5	0.28–0.88	0.016	1.1
Pear body shape	0.5	0.44–0.66	< 0.001	1.8

*Notes* PR = prevalence ratio; CI = confidence interval; VIF: variance inflation factor. Menarche was removed from the model due to its variance inflation factors (VIF) exceeding 5, specifically 14.2. Brier Score: 0.038. Area under ROC curve: 0.647

Table 5 displays the independent factors associated with chronic pelvic pain as a whole, considering it without isolating the subtypes. We observed an overlap of associations, except for subfertility, which emerged as an additional factor positively associated with the outcome.

**Table 5:** Features independently associated with all types of chronic pelvic pain in the population predominantly indigenous of Otavalo, Ecuador

Features	PR	95% CI	adjusted *p*-value	VIF
Subfertility	2.4	1.29–4.49	0.006	1.0
Muscle/joint pain	1.4	0.96–2.10	0.083	1.0
Intestinal symptoms	1.3	1.17–1.47	< 0.001	1.6
Urinary symptoms	1.3	1.13–1.44	< 0.001	1.5
Catastrophizing	1.0	1.02–1.03	< 0.001	1.7
Unmixed ancestry	0.8	0.71–0.86	< 0.001	1.6
Hormone usage	0.8	0.62–1.03	0.090	1.1
Previous pregnancy	0.6	0.52–0.63	< 0.001	2.1
Pear body shape	0.5	0.44–0.54	< 0.001	1.6

*Notes* PR = prevalence ratio; CI = confidence interval; VIF: variance inflation factor. Age was removed from the model due to its respective variance inflation factors (VIF) exceeding 5, specifically 7.1. Brier Score: 0.187. Area under ROC curve: 0.731

Supplementary Table 2 shows the full models for each one of the primary outcomes.

## Discussion

The results of this study revealed that within this population, the prevalence of chronic pelvic pain is high, with primary dysmenorrhea being the predominant subtype. More than a quarter of women, up to two years after menarche, experienced primary dysmenorrhea, approximately 10% reported non-cyclical pelvic pain, and almost 4% suffered from dyspareunia. All chronic pain conditions were independently associated with one another. Considering that primary dysmenorrhea occurs temporally before the others, although it cannot be definitively confirmed in this study’s design, it is plausible to hypothesise about a potential causal or facilitating effect of primary dysmenorrhea on other chronic pains, a notion already discussed in the literature [18].

The prevalence of primary dysmenorrhea identified falls within the range reported worldwide, but interestingly, it is nearly double the rate we previously identified in the non-indigenous population living in an urban area in the capital of Ecuador [54]. In that population, the observed rate of hormonal contraceptive use was considered low, at around 25%, while in this indigenous population, the usage rate is only 5.5%, ranging from 3% among those with primary dysmenorrhea to 6% among those without this condition. We believe that this difference can be due to cultural and religious reasons. Considering that these medications are associated with a significant improvement in dysmenorrhea symptoms [55], the low frequency of usage, in our view, may be a crucial factor contributing to the increased reporting of menstrual pain by these women.

On the other hand, there is a lower prevalence of primary dysmenorrhea among indigenous women without a history of interbreeding in the family. Although our study cannot deeply discuss this difference, we can propose at least two hypotheses. The first is that there may be a specific racial and/or genomic characteristic of this population. However, this is purely speculative, as genomic data on indigenous populations are still limited [56]. The second hypothesis we consider is socio-cultural significance. Menstruation in the indigenous community that maintains its deep-rooted beliefs is often characterised as “private women’s business.” It is sometimes seen as a sign of impurity. Stigma, secrecy, and shame associated with discussing menstruation can reduce symptom reporting among indigenous women with more conservative cultural values and taboos [57]. This may make dysmenorrhea less likely to be reported in this group.

Another point that captures our attention is the association of primary dysmenorrhea with systemic arterial hypertension, although the confidence interval of the prevalence ratio has included the null value. The link with adverse cardiovascular events has already been identified by other researchers [20, 58], and this may perhaps be attributed to a systemic inflammatory status observed in these women, which, however, requires more detailed evaluation [59]. Studies have shown an association between cardiovascular events not only with a higher amount of body fat but also with its distribution [60, 61]. The “pear” shape, with a more homogeneous distribution of fat tissue primarily on the hips, has been associated with this [62].

In parallel, the relationship between BMI and dysmenorrhea is controversial. Longitudinal studies with large cohorts have shown evidence of a U-shaped relationship between these conditions [63], suggesting a more significant connection with body constitution than weight itself. Finally, a large British cohort has demonstrated significant associations between body shape and inflammatory and metabolic biomarkers [64].

In our study, the prevalence of non-cyclical pelvic pain was similar to that observed in Latin American countries such as Brazil, where it’s close to 10% [65, 66], and in urban communities of Ecuador, where it is 8.9% [54]. It was positively associated with various other painful conditions, reinforcing the link between the condition and nociplastia [67], and perhaps reflecting the clinical expression of central sensitization that commonly occurs in this group of patients [68]. These findings are also supported by the apparent protective effect identified in the practice of physical exercise and non-cyclical pelvic pain. Recent literature has shown that physical exercise can strengthen the modulation promoted by the central nervous system [69, 70], reducing pain sensitization [71], and there is a direct inverse relationship between measures of physical activity and chronic pain levels [72].

The observed prevalence of dyspareunia in this population was significantly lower than that previously reported in the urban population of Ecuador [54]. The exact reason for this difference remains uncertain. Regardless of the low prevalence of dyspareunia in the studied population, what is equally remarkable is the fact that virtually all the women who reported pain during sexual intercourse did not discontinue intercourse for that reason. Taken together, we believe that this finding could be attributed to the patriarchal structure of indigenous society, where “male” attitudes prevail, exacerbating the social and biological vulnerability to which indigenous women are historically subjected. They have often been tied to familial and communal roles, hindering their ability to express their desires and preferences (http://repositorio.utn.edu.ec/handle/123456789/6165*)* [73].

Despite the absence of affirmative responses regarding the presence of intrauterine infections, 66.3% of the women reported frequent and concurrent urinary symptoms, which could potentially be linked to infectious processes secondary to *Neisseria gonorrhoeae* and *Chlamydia trachomatis*, causative bacterial agents of urethritis and pelvic inflammatory disease [74]. An underdiagnosis of pelvic inflammatory disease might also explain the association with previous pregnancy losses, as there is a connection between intrauterine infection and abortions [75]. The findings concerning urinary discomfort contrast with those related to intrauterine infections, which exhibit a notably low prevalence.

Additionally, we identified that subfertility is an independent factor associated with pelvic pain overall. This may reflect the presence of an underlying condition or disease such as endometriosis or pelvic inflammatory disease. However, unfortunately, this could not be accurately assessed in this study. The knowledge of the interviewed women about these subjects was limited, and only a very small proportion answered the related questions confidently.

We did not observe any association between pain and psychological symptoms, smoking, alcoholism, or violence, as we had observed in other studies conducted in Ecuador and Brazil. One possible justification for this could be the low reported prevalence of these conditions in this specific community, which is close to or less than 1% for each.

### Strengthens and limitations

Our study’s strength lies in the inclusion of a large and representative cohort of the Kichwa indigenous population. Furthermore, it was conducted with meticulous methodological rigour, granting it robust inferential power. However, there are certain limitations associated with both the characteristics of the population and the analysis itself.

Ethnic self-identification, defined as “the right of every person to freely and voluntarily decide whether or not to belong to a nationality or people” may have also altered the population distribution of the women studied, as each individual can choose to identify themselves accurately or erroneously with a particular nationality or people, even if they do not genuinely belong.

The results of this study may also be subject to biases primarily related to the indigenous and Andean worldviews. These worldviews, shaped by beliefs, values, and knowledge systems, play a pivotal role in the social life of these human groups and define their cultural identity. It is crucial to emphasise the social and biological vulnerability of indigenous women, who unfortunately remain entrenched in economic, social, and cultural inequalities, where patriarchal social structures persist. Even though all our interviewers were women representing the local indigenous community, which facilitated the feasibility of the research, some topics are still considered taboo, particularly concerning sexual activity, illicit substance use, tobacco, alcohol, psychological symptoms, and violence. We believe this might have influenced the identification of independently associated factors and, in some way, hindered the formulation of education and healthcare policies tailored to indigenous women, especially with regard to dyspareunia.

Regarding the analysis, certain aspects are inherent to logistic regression models. For our study, the backward stepwise approach proved to be the most suitable model for the data. It may be influenced by the relationship between the number of candidate variables and the sample size, but this was not a concern given our relatively large cohort. Including all variables would add significant complexity and could potentially compromise the model’s generalizability. To mitigate this, we conducted a correlogram and aimed to avoid including highly correlated variables in the simulated models. Nonetheless, this was done judiciously to prevent the premature exclusion of relevant variables.

It is also challenging to ensure that all potential combinations of predictors have been tested. The significance of the p-value does not always equate to clinical relevance, making the interpretation of the effect (in this case, prevalence ratio) crucial. Moreover, it is impossible to establish a causal relationship between the outcomes and associated variables, which is a limitation inherent to cross-sectional studies.

On the other hand, backward elimination allows for the advantage of initially considering the effects of all variables simultaneously, which is especially important in cases of potential collinearity, as mentioned earlier. Other factors that balance its limitations include its ease of application, objectivity, reproducibility, interpretability, and the enhanced generalisation achieved by reducing the number of predictor variables.

## Conclusions

The prevalence of chronic pelvic pain overall was high, 33.0%. Primary dysmenorrhea and non-cyclic chronic pelvic pain in Kichwa women from Otavalo was notably high, while the frequency of reported dyspareunia was comparatively low, at 26.6%, 8.9%, and 3.9% respectively. There exists a direct correlation between the outcomes of all forms of chronic pelvic pain. We have identified a significant association between primary dysmenorrhoea and conditions related to inflammatory and/or systemic metabolic disorders, warranting special attention. Similarly, a connection was established between non-cyclical pelvic pain and signs/symptoms that could collectively signify central sensitization and nociplastia. Furthermore, dyspareunia was additionally linked to conditions frequently associated with infections, which, in turn, were not reported by the participants. Subfertility was associated with pelvic pain overall, but not specifically with individual subtypes. Taking into account the temporal relationship between the conditions, the study suggested a potential causal association between primary dysmenorrhea and the development of dyspareunia and acyclic pelvic pain in the future. We consider welcoming and offering additional care opportunities to these women to be essential. In conclusion, considering that the concepts of health and illness in this population differ from typical Western ideals, public policies should take into account the provision of education and healthcare services in alignment with the values and principles of the local community.

### Electronic supplementary material

Below is the link to the electronic supplementary material.

Supplementary Material 1

## Data Availability

The data that support the findings of this study are available from the corresponding author, [OBPN], upon reasonable request.

## Abbreviations

AIC: Akaike information criteria
BIC: Bayesian information criteria
BMI: Body mass index
COVID-19: Coronavirus disease 2019
CPP: Chronic pelvic pain
IASP: International Association for the Study of Pain
RCOG: Royal College of Obstetricians and Gynaecologists
REDCap: Research Electronic Data Capture
ROC: Receiver operating characteristics
SOP: Standard operating procedures
VIF: Variance inflation factor
WERF EPHect: World Endometriosis Research Foundation Endometriosis Phenome and Biobanking Harmonisation Project

## References

[1] Ayorinde AA, Bhattacharya S, Druce KL, Jones GT, Macfarlane GJ (2017). Chronic pelvic pain in women of reproductive and post-reproductive age: a population-based study. Eur J Pain Lond Engl março de.

[2] Ahangari A (2014). Prevalence of chronic pelvic pain among women: an updated review. Pain Physician.

[3] Latthe P, Latthe M, Say L, Gülmezoglu M, Khan KS (2006). WHO systematic review of prevalence of chronic pelvic pain: a neglected reproductive health morbidity. BMC Public Health 6 de dezembro de.

[4] Romão APMS, Gorayeb R, Romão GS, Poli-Neto OB, Dos Reis FJC, Rosa-E-Silva JC et al. Chronic pelvic pain: multifactorial influences. J Eval Clin Pract. 2011;17(6). 10.1111/j.1365-2753.2010.01485.x 20630008

[5] Grace V, Zondervan K (2006). Chronic Pelvic Pain in women in New Zealand: Comparative Well-Being, Comorbidity, and impact on work and other activities. Health Care Women Int agosto de.

[6] Tripoli TM, Sato H, Sartori MG, de Araujo FF, Girão MJBC, Schor E (2011). Evaluation of quality of life and sexual satisfaction in women suffering from chronic pelvic pain with or without endometriosis. J Sex Med.

[7] Stones RW, Price C. Health services for women with chronic pelvic pain. J R Soc Med. 1^o^ de novembro de. 2002;95(11):531–5. 10.1258/jrsm.95.11.531 PMC1279247 12411615

[8] Chen I, Thavorn K, Shen M, Goddard Y, Yong P, MacRae GS (2017). Hospital-associated costs of Chronic Pelvic Pain in Canada: a Population-based descriptive study. J Obstet Gynaecol Can março de.

[9] Hooker AB, van Moorst BR, van Haarst EP, van Ootegehem NA, van Dijken DK, Heres MH. Chronic pelvic pain: evaluation of the epidemiology, baseline demographics, and clinical variables via a prospective and multidisciplinary approach. Clin Exp Obstet Gynecol. 1^o^ de janeiro de. 2013;40(4):492–8. 24597241

[10] Latthe PM, Champaneria R, Dysmenorrhoea (2011). BMJ Clin Evid 21 de fevereiro de.

[11] Fang X, Liu H, Wang M, Wang G (2023). Scientific knowledge graph of Dysmenorrhea: a bibliometric analysis from 2001 to 2021. J Pain Res.

[12] MacGregor B, Allaire C, Bedaiwy MA, Yong PJ, Bougie O (2023). Disease Burden of Dysmenorrhea: impact on Life Course potential. Int J Womens Health.

[13] Unsal A, Ayranci U, Tozun M, Arslan G, Calik E (2010). Prevalence of dysmenorrhea and its effect on quality of life among a group of female university students. Ups J Med Sci maio de.

[14] Sahin N, Kasap B, Kirli U, Yeniceri N, Topal Y (2018). Assessment of anxiety-depression levels and perceptions of quality of life in adolescents with dysmenorrhea. Reprod Health 26 de janeiro de.

[15] Ishikura IA, Hachul H, Pires GN, Tufik S, Andersen ML. The impact of primary dysmenorrhea on sleep and the consequences for adolescent academic performance. J Clin Sleep Med JCSM off Publ Am Acad Sleep Med. 15 de março de. 2020;16(3):467–8. 10.5664/jcsm.8238 PMC7075086 31992418

[16] Kabukçu C, Kabukçu Başay B, Başay Ö (2021). Primary dysmenorrhea in adolescents: Association with attention deficit hyperactivity disorder and psychological symptoms. Taiwan J Obstet Gynecol março de.

[17] Armour M, Parry K, Manohar N, Holmes K, Ferfolja T, Curry C (2019). The prevalence and academic impact of Dysmenorrhea in 21,573 Young women: a systematic review and Meta-analysis. J Womens Health 2002 agosto de.

[18] Li R, Li B, Kreher DA, Benjamin AR, Gubbels A, Smith SM (2020). Association between dysmenorrhea and chronic pain: a systematic review and meta-analysis of population-based studies. Am J Obstet Gynecol setembro de.

[19] Li R, Kreher DA, Jusko TA, Chapman BP, Bonham AD, Seplaki CL (2021). Prospective Association between Dysmenorrhea and Chronic Pain Development in Community-Dwelling women. J Pain setembro de.

[20] Yeh CH, Muo CH, Sung FC, Yen PS (2022). Risk of Ischemic Heart Disease Associated with primary dysmenorrhea: a Population-based Retrospective Cohort Study. J Pers Med 29 de setembro de.

[21] Hayes RD, Bennett CM, Fairley CK, Dennerstein L (2006). What can prevalence studies tell us about female sexual difficulty and dysfunction?. J Sex Med julho de.

[22] Mitchell KR, Geary R, Graham CA, Datta J, Wellings K, Sonnenberg P (2017). Painful sex (dyspareunia) in women: prevalence and associated factors in a British population probability survey. BJOG Int J Obstet Gynaecol outubro de.

[23] Pazmany E, Bergeron S, Van Oudenhove L, Verhaeghe J, Enzlin P (2013). Aspects of sexual self-schema in premenopausal women with dyspareunia: associations with pain, sexual function, and sexual distress. J Sex Med setembro de.

[24] Payne KA, Binik YM, Amsel R, Khalifé S (2005). When sex hurts, anxiety and fear orient attention towards pain. Eur J Pain Lond Engl agosto de.

[25] MacNeill C, Dyspareunia (2006). Obstet Gynecol Clin North Am dezembro de.

[26] Facchin F, Buggio L, Dridi D, Barbara G, Vercellini P. The subjective experience of Dyspareunia in Women with endometriosis: a systematic review with narrative synthesis of qualitative research. Int J Environ Res Public Health. 18 de novembro de. 2021;18(22):12112. 10.3390/ijerph182212112 PMC8623407 34831868

[27] Landry T, Bergeron S (2011). Biopsychosocial factors associated with dyspareunia in a community sample of adolescent girls. Arch Sex Behav outubro de.

[28] Lemieux AJ, Bergeron S, Steben M, Lambert B (2013). Do romantic partners’ responses to entry dyspareunia affect women’s experience of pain? The roles of catastrophizing and self-efficacy. J Sex Med setembro de.

[29] Jimenez N, Garroutte E, Kundu A, Morales L, Buchwald D (2011). A review of the experience, epidemiology, and management of pain among American Indian, Alaska Native, and Aboriginal Canadian peoples. J Pain maio de.

[30] Miller ET, Abu-Alhaija DM (2019). Cultural influences on Pain Perception and Management. Pain Manag Nurs off J am soc Pain Manag nurses. junho de.

[31] Jagger RG, Woolley SM, Savio L (2004). Signs and symptoms of temporomandibular disorders in Ecuadorian indians. J Oral Rehabil Abril De.

[32] Carod-Artal FJ, Vázquez-Cabrera C (2007). An anthropological study about headache and migraine in native cultures from Central and South America. Headache junho de.

[33] Darghouth S, Pedersen D, Bibeau G, Rousseau C (2006). Painful languages of the body: experiences of headache among women in two Peruvian communities. Cult Med Psychiatry setembro de.

[34] Barbosa de Moraes E, Dal Fabbro DR, Bernardes de Oliveira L (2021). Ribeiro Leão E. Pain Management of Amazon Indigenous peoples: A Community-based study. J Pain Res.

[35] Dawson AZ, Walker RJ, Campbell JA, Davidson TM, Egede LE (2020). Telehealth and indigenous populations around the world: a systematic review on current modalities for physical and mental health. mHealth.

[36] Durie MH (2003). The health of indigenous peoples. BMJ 8 de março de.

[37] Horrill T, McMillan DE, Schultz ASH, Thompson G (2018). Understanding access to healthcare among indigenous peoples: a comparative analysis of biomedical and postcolonial perspectives. Nurs Inq julho de.

[38] Flores-Ramírez R, Berumen-Rodríguez AA, Martínez-Castillo MA, Alcántara-Quintana LE, Díaz-Barriga F (2021). Díaz De León-Martínez L. A review of environmental risks and vulnerability factors of indigenous populations from Latin America and the Caribbean in the face of the COVID–19. Glob Public Health julho de.

[39] Latimer M, Rudderham S, Lethbridge L, MacLeod E, Harman K, Sylliboy JR et al. Occurrence of and referral to specialists for pain-related diagnoses in First Nations and non-first nations children and youth. CMAJ Can Med Assoc J J Assoc Medicale Can. 10 de dezembro de. 2018;190(49):E1434–40. 10.1503/cmaj.180198 PMC6279448 30530610

[40] Mittinty MM, McNeil DW, Jamieson LM (2018). Limited evidence to measure the impact of chronic pain on health outcomes of indigenous people. J Psychosom Res Abril De.

[41] de Oliveira PR, Pereira LV, da Silva Carvalho Vila V, Guimarães Lemes A, da Rocha EM, Ferreira AB (2023). Pain management in indigenous and tribal peoples: a scoping review protocol. BMJ Open 9 de agosto de.

[42] Rios-Quituizaca P, Gatica-Domínguez G, Nambiar D, Santos JLF, Barros AJD (2022). Ethnic inequalities in reproductive, maternal, newborn and child health interventions in Ecuador: a study of the 2004 and 2012 national surveys. EClinicalMedicine março de.

[43] Etxabe AM. Comunidad Campesina Kichwa Y cosmovisión indíguena en El Ecuador. Coop E Econ Soc. 1999;(21–22):147–64.

[44] Incayawar M, Saucier JF. Exploring pain in the Andes – learning from the Quichua (Inca) people experience. Postgrad Med. 4 de julho de. 2015;127(4):368–75. 10.1080/00325481.2015.1015395 25697331

[45] Patridge EF, Bardyn TP (2018). Research Electronic Data Capture (REDCap). J Med Libr Assoc JMLA janeiro de.

[46] Becker CM, Laufer MR, Stratton P, Hummelshoj L, Missmer SA, Zondervan KT (2014). World Endometriosis Research Foundation Endometriosis Phenome and Biobanking Harmonisation Project: I. Surgical phenotype data collection in endometriosis research. Fertil Steril novembro de.

[47] Fassbender A, Rahmioglu N, Vitonis AF, Viganò P, Giudice LC, D’Hooghe TM (2014). World Endometriosis Research Foundation Endometriosis Phenome and Biobanking Harmonisation Project: IV. Tissue collection, processing, and storage in endometriosis research. Fertil Steril novembro de.

[48] Rahmioglu N, Fassbender A, Vitonis AF, Tworoger SS, Hummelshoj L, D’Hooghe TM (2014). World Endometriosis Research Foundation Endometriosis Phenome and Biobanking Harmonization Project: III. Fluid biospecimen collection, processing, and storage in endometriosis research. Fertil Steril novembro de.

[49] Vitonis AFAF, Vincent K, Rahmioglu N, Fassbender A, Buck Louis GMGM, Hummelshoj L (2014). World Endometriosis Research Foundation Endometriosis Phenome and biobanking harmonization project: II. Clinical and covariate phenotype data collection in endometriosis research. Fertil Steril novembro de.

[50] Harris PA, Taylor R, Thielke R, Payne J, Gonzalez N, Conde JG. Research electronic data capture (REDCap)—A metadata-driven methodology and workflow process for providing translational research informatics support. J Biomed Inf 1^o^ De Abril De. 2009;42(2):377–81. 10.1016/j.jbi.2008.08.010 PMC2700030 18929686

[51] Harris PA, Taylor R, Minor BL, Elliott V, Fernandez M, O’Neal L, et al. The REDCap consortium: building an international community of software platform partners. J Biomed Inf 1^o^ De julho de. 2019;95:103208. 10.1016/j.jbi.2019.103208 PMC7254481 31078660

[52] Pourhoseingholi MA, Vahedi M, Rahimzadeh M (2013). Sample size calculation in medical studies. Gastroenterol Hepatol Bed Bench.

[53] García Campayo J, Rodero B, Alda M, Sobradiel N, Montero J, Moreno S. [Validation of the Spanish version of the Pain Catastrophizing Scale in Fibromyalgia]. Med Clin (Barc). 18 de outubro de. 2008;131(13):487–92. 10.1157/13127277 19007576

[54] de Rosero LMV, Mazin CY, Nogueira SC, Vargas-Costales AA, Rosa-E-Silva JA, Candido-Dos-Reis JC (2022). Prevalence of chronic pelvic pain and primary dysmenorrhea in women of reproductive age in Ecuador. BMC Womens Health 2 de setembro de.

[55] Schroll JB, Black AY, Farquhar C, Chen I. Combined oral contraceptive pill for primary dysmenorrhoea. Cochrane Database Syst Rev. 31 de julho de. 2023;7(7):CD002120. 10.1002/14651858.CD002120.pub4 PMC10388393 37523477

[56] De Oliveira TC, Secolin R, Lopes-Cendes I (2023). A review of ancestrality and admixture in Latin America and the caribbean focusing on native American and African descendant populations. Front Genet.

[57] Ciccia D, Doyle AK, Ng CHM, Armour M. Indigenous Peoples’ Experience and Understanding of Menstrual and Gynecological Health in Australia, Canada and New Zealand: A Scoping Review. Int J Environ Res Public Health. 7 de julho de. 2023;20(13):6321. 10.3390/ijerph20136321 PMC10341312 37444168

[58] Chung HF, Ferreira I, Mishra GD (2021). The association between menstrual symptoms and hypertension among young women: a prospective longitudinal study. Maturitas janeiro de.

[59] Szmidt MK, Granda D, Sicinska E, Kaluza J. Primary dysmenorrhea in relation to oxidative stress and antioxidant status: a systematic review of case-control studies. Antioxid Basel Switz. 15 de outubro de. 2020;9(10):994. 10.3390/antiox9100994 PMC7602455 33076228

[60] Wang S, Liu Y, Li F, Jia H, Liu L, Xue F. A novel quantitative body shape score for detecting association between obesity and hypertension in China. BMC Public Health. 17 de janeiro de. 2015;15:7. 10.1186/s12889-014-1334-5 PMC4308906 25595192

[61] Oh CM, Park JH, Chung HS, Yu JM, Chung W, Kang JG et al. Effect of body shape on the development of cardiovascular disease in individuals with metabolically healthy obesity. Med (Baltim). 18 de setembro de. 2020;99(38):e22036. 10.1097/MD.0000000000022036 PMC7505363 32957321

[62] Karastergiou K, Smith SR, Greenberg AS, Fried SK (2012). Sex differences in human adipose tissues - the biology of pear shape. Biol Sex Differ 31 de maio de.

[63] Ju H, Jones M, Mishra GD (2015). A U-Shaped relationship between body Mass Index and Dysmenorrhea: a longitudinal study. PLoS ONE.

[64] Christakoudi S, Riboli E, Evangelou E, Tsilidis KK (2022). Associations of body shape index (ABSI) and hip index with liver, metabolic, and inflammatory biomarkers in the UK Biobank cohort. Sci Rep 25 de maio de.

[65] Silva GP, de OG da, Nascimento AL do, Michelazzo D, AJ FF, R MGMG, S JCRE, et al. High prevalence of chronic pelvic pain in women in Ribeirão Preto, Brazil and direct association with abdominal surgery. Clin Sao Paulo Braz. 2011;66(8):1307–12. 10.1590/S1807-59322011000800001 PMC3161204 21915476

[66] Coelho LSCSC, Brito LMOMO, Chein MBCBC, Mascarenhas TSS, Costa JPLPL, Nogueira AAA (2014). Prevalence and conditions associated with chronic pelvic pain in women from São Luís, Brazil. Braz J Med Biol Res setembro de.

[67] Till SR, Schrepf A, Clauw DJ, Harte SE, Williams DA, As-Sanie S (2023). Association between Nociplastic Pain and Pain Severity and Impact in Women with Chronic Pelvic Pain. J Pain agosto de.

[68] Levesque A, Riant T, Ploteau S, Rigaud J, Labat JJ. Clinical criteria of central sensitization in chronic pelvic and perineal pain (Convergences PP Criteria): Elaboration of a clinical evaluation tool based on formal expert consensus. Pain Med U S. 1^o^ de outubro de. 2018;19(10):2009–15. 10.1093/pm/pny030 PMC7372934 29522121

[69] Ellingson LD, Koltyn KF, Kim JS, Cook DB (2014). Does exercise induce hypoalgesia through conditioned pain modulation? Psychophysiology. março de.

[70] Ferro Moura Franco K, Lenoir D, Dos Santos Franco YR, Jandre Reis FJ, Nunes Cabral CM, Meeus M (2021). Prescription of exercises for the treatment of chronic pain along the continuum of nociplastic pain: a systematic review with meta-analysis. Eur J Pain Lond Engl janeiro de.

[71] Tan L, Cicuttini FM, Fairley J, Romero L, Estee M, Hussain SM (2022). Does aerobic exercise effect pain sensitisation in individuals with musculoskeletal pain? A systematic review. BMC Musculoskelet Disord 3 de fevereiro de.

[72] Fjeld MK, Årnes AP, Engdahl B, Morseth B, Hopstock LA, Horsch A, et al. Consistent pattern between physical activity measures and chronic pain levels: the Tromsø Study 2015 to 2016. Pain 1^o^ De Abril De. 2023;164(4):838–47. 10.1097/j.pain.0000000000002773 PMC10026831 36083173

[73] Segarra J, Argudo MVF, Espinoza E, del Crespo CP, Brito B, Cisneros DAJ. MAC, Percepciones sobre la salud sexual y reproductiva de las mujeres indígenas Kichwas y Shuaras. Ecuador, 2016. Em 2016 [citado 6 de novembro de 2023]. Disponível em: https://www.semanticscholar.org/paper/Percepciones-sobre-la-salud-sexual-y-reproductiva-y-Segarra-Argudo/fbfbb450d9d5186ed361bff2dd64a0c5b2518d60.

[74] Darville T (2021). Pelvic inflammatory Disease due to Neisseria gonorrhoeae and Chlamydia trachomatis: Immune Evasion mechanisms and pathogenic Disease pathways. J Infect Dis 16 de agosto de.

[75] Patel SV, Baxi RK, Kotecha PV, Mazumdar VS, Mehta KG, Diwanji M (2010). Association between pelvic inflammatory disease and abortions. Indian J Sex Transm Dis AIDS.

