# Supplementary material for: Prevalence of chronic pelvic pain and associated factors among indigenous women of reproductive age in Ecuador
Source: BMC Womens Health. 2024 Jul 4;24:388. doi: 10.1186/s12905-024-03189-7 (PMC11223279; doi:10.1186/s12905-024-03189-7)
Supplement: Supplementary file 1 — Supplementary Material 1 [file 12905_2024_3189_MOESM1_ESM.docx]

**Supplementary Material**

Supplementary Table 1. Stratified proportional sampling by parishes of the Otavalo canton.

| Parishes | Population (2020) | Sample planned  number (%) | Sample carried out  (number, %) |
| --- | --- | --- | --- |
| San Pedro de Pataquí | 269 | 6 (0.26) | 6 (0.25) |
| Selva Alegre | 1,600 | 37 (1.53) | 37 (1.52) |
| Dr. Miguel Egas | 4,883 | 112 (4.66) | 105 (4.32) |
| San Rafael | 5,421 | 124 (5.17) | 125 (5.15) |
| González Suárez | 5,630 | 129 (5.37) | 127 (5.23) |
| Eugenio Espejo | 7,357 | 168 (7.02) | 179 (7.37) |
| San José de Quichinche | 8,476 | 194 (8.08) | 197 (8.11) |
| San Juan de Ilumán | 8,584 | 197 (8.19) | 201 (8.28) |
| San Pablo del Lago | 9,901 | 227 (9.44) | 229 (9.43) |
| Otavalo | 52,753 | 1,208 (50.30) | 1,223 (50.35) |
| Total | 104,874 | 2,401 | 2,429 |

Supplementary Table 2. Logistic regression including all variables for each of the primary outcomes (full model).

| Variables |  | Chronic pelvic pain | | Primary dysmenorrhoea | | Non-cyclic pelvic pain | | Dyspareunia | |
| --- | --- | --- | --- | --- | --- | --- | --- | --- | --- |
|  |  | PR [95% CI] | p-value | PR [95% CI] | p-value | PR [95% CI] | p-value | PR [95% CI] | p-value |
| Age (years) | | 0.98 [0.97-0.99] | .001 | 0.97 [0.96-0.98] | <.001 | 1.00 [0.99-1.01] | .895 | 1.01 [0.99-1.03] | .323 |
| Education level (minimum) | | 0.95 [0.82-1.11] | .534 | 0.88 [0.74-1.04] | .141 | 1.07 [0.88-1.30] | .516 | 0.80 [0.58-1.10] | .167 |
| Obesity (BMI>30) | | 1.06 [0.88-1.27] | .550 | 0.97 [0.79-1.20] | .806 | 1.16 [0.93-1.46] | .180 | 1.05 [0.75-1.47] | .770 |
| Exercise (>2h/day) | | 1.05 [0.93-1.17] | .454 | 1.14 [1.01-1.29] | .038 | 0.84 [0.72-0.98] | .025 | 1.02 [0.80-1.30] | .879 |
| Body type (pear) | | 0.51 [0.46-0.57] | <.001 | 0.53 [0.47-0.60] | <.001 | 0.71 [0.61-0.83] | <.001 | 0.75 [0.59-0.95] | .020 |
| Catastrophizing (score) | | 1.02 [1.02-1.03] | <.001 | 1.02 [1.01-1.03] | <.001 | 1.01 [1.00-1.02] | .011 | 1.01 [1.00-1.02] | .153 |
| Indigenous ethnicity | | 0.85 [0.76-0.95] | .006 | 0.83 [0.73-0.94] | .003 | 0.93 [0.80-1.09] | .398 | 0.96 [0.76-1.22] | .751 |
| Menarche (years) | | 1.00 [0.98-1.02] | .771 | 1.02 [1.00-1.04] | .126 | 0.87 [0.85-0.90] | <.001 | 0.82 [0.78-0.85] | <.001 |
| Hormone usage (contraceptive) | | 0.78 [0.60-1.02] | .065 | 0.76 [0.57-1.01] | .055 | 0.97 [0.70-1.36] | .872 | 0.76 [0.43-1.35] | .348 |
| Menstrual irregularity | | 1.19 [1.03-1.39] | .021 | 1.05 [0.90-1.23] | .534 | 1.12 [0.92-1.37] | .260 | 1.19 [0.90-1.59] | .229 |
| Prolonged periods | | 1.46 [0.96-2.22] | .075 | 1.04 [0.66-1.62] | .879 | 1.46 [0.92-2.33] | .112 | 0.95 [.045-1.96] | .880 |
| Heavy periods | | 0.99 [0.86-1.12] | .822 | 1.01 [0.87-1.16] | .942 | 0.97 [0.82-1.16] | .760 | 0.99 [0.76-1.29] | .933 |
| Cycle length (<24 days) | | 1.10 [0.82-1.47] | .530 | 1.24 [0.92-1.67] | .154 | 1.09 [0.74-1.58] | .669 | 0.74 [0.39-1.41] | .358 |
| Cycle length (>38 days) | | 0.79 [0.63-0.99] | .044 | 0.79 [0.62-1.01] | .060 | 1.05 [0.79-1.40] | .720 | 1.06 [0.70-1.62] | .780 |
| Ever sexually active | | 1.19 [0.99-1.43] | .058 | 1.14 [0.95-1.37] | .150 | 1.12 [0.84-1.49] | .442 | — | — |
| Previous pregnancy | | 0.74 [0.62-0.89] | .001 | 0.75 [0.62-0.92] | .005 | 1.14 [0.88-1.47] | .308 | 1.19 [0.82-1.73] | .350 |
| Subfertility | | 2.07 [1.08-3.98] | .029 | 1.43 [0.76-2.69] | .263 | 1.04 [0.53-2.05] | .909 | 1.98 [0.94-4.18] | .074 |
| Teenage pregnancy | | 0.88 [0.76-1.03] | .102 | 0.84 [0.71-0.99] | .038 | 1.04 [0.86-1.25] | .695 | 0.95 [0.73-1.25] | .726 |
| Previous cesarean section | | 1.02 [0.79-1.32] | .870 | 1.10 [0.83-1.45] | .507 | 1.16 [0.86-1.58] | .337 | 0.72 [0.45-1.16] | .176 |
| Previous pregnancy loss | | 1.33 [0.98-1.81] | .068 | 0.84 [0.58-1.20] | .329 | 1.29 [0.90-1.84] | .162 | 1.81 [1.19-2.77] | .006 |
| Alcoholism | | 0.73 [0.38-1.41] | .351 | 0.81 [0.41-1.60] | .537 | 0.82 [0.32-2.09] | .672 | 0.00 [0.00-inf] | .999 |
| Smoking | | 1.25 [0.54-2.92] | .604 | 1.36 [0.57-3.29] | .488 | 0.69 [0.19-2.58] | .584 | 2.08 [0.55-7.77] | .278 |
| Anxiety | | 1.13 [0.48-2.63] | .782 | 1.07 [0.44-2.57] | .882 | 2.06 [0.76-2.55] | .154 | 0.00 [0.00-inf] | .999 |
| Depression | | — | — | — | — | — | — | — | — |
| Diabetes | | 1.20 [0.58-2.49] | .631 | 1.37 [0.62-3.02] | .440 | 0.64 [0.21-1.91] | .421 | 1.58 [0.53-4.70] | .414 |
| Hypertension | | 1.46 [0.81-2.65] | .210 | 2.38 [1.30-4.35] | .005 | 0.93 [0.46-1.89] | .849 | 0.00 [0.00-inf] | .999 |
| Intestinal symptoms | | 1.34 [1.19-1.51] | <.001 | 1.37 [1.21-1.56] | <.001 | 1.02 [0.87-1.21] | .786 | 1.02 [0.80-1.30] | .863 |
| Urinary symptoms | | 1.34 [1.18-1.52] | <.001 | 1.14 [1.00-1.31] | .052 | 1.22 [1.03-1.44] | .018 | 1.79 [1.41-2.27] | <.001 |
| Migraine | | 0.89 [0.52-1.55] | .686 | 0.96 [0.53-1.72] | .881 | 1.37 [0.74-2.55] | .320 | 0.99 [0.33-2.96] | .992 |
| Previous surgery | | 1.11 [0.94-1.33] | .223 | 1.11 [0.92-1.35] | .285 | 1.04 [0.84-1.30] | .715 | 1.13 [0.83-1.54] | .439 |
| Primary dysmenorrhoea | | — | — | — | — | 1.41 [1.18-1.68] | <.001 | 1.75 [1.35-2.27] | <.001 |
| Non-cyclic pelvic pain | | — | — | 1.45 [1.19-1.78] | <.001 | — | — | 1.53 [1.15-2.05] | .004 |
| Dyspareunia | | — | — | 1.75 [1.27-2.40] | .001 | 1.74 [1.26-2.41] | .001 | — | — |
| Familial history of pelvic pain | | 0.95 [0.76-1.19] | .653 | 0.84 [0.66-1.07] | .153 | 1.16 [0.88-1.52] | .300 | 0.97 [0.63-1.48] | .879 |
| Low back pain | | 0.88 [0.67-1.16] | .373 | 0.82 [0.60-1.13] | .230 | 1.13 [0.81-1.58] | .484 | 0.51 [0.20-1.32] | .167 |
| Muscle/joint pain | | 1.51 [0.98-2.33] | .060 | 1.49 [0.94-2.37] | .091 | 1.58 [0.98-2.54] | .060 | 0.91 [0.33-2.49] | .859 |
| Pain at ovulation | | 1.22 [0.82-1. | .325 | 1.10 [0.71-1.71] | .666 | 1.50 [0.95-2.36] | .079 | 0.00 [0.00-inf] | .999 |

Notes: PR = prevalence ratio; CI = confidence interval
